# Prostaglandin E2-Induced COX-2 Expressions via EP2 and EP4 Signaling Pathways in Human LoVo Colon Cancer Cells

**DOI:** 10.3390/ijms18061132

**Published:** 2017-05-25

**Authors:** Hsi-Hsien Hsu, Yueh-Min Lin, Chia-Yao Shen, Marthandam Asokan Shibu, Shin-Yi Li, Sheng-Huang Chang, Chien-Chung Lin, Ray-Jade Chen, Vijaya Padma Viswanadha, Hui-Nung Shih, Chih-Yang Huang

**Affiliations:** 1 Division of Colorectal Surgery, Mackay Memorial Hospital, Freshwater 25160, Taiwan; hsu5936@ms3.hinet.net (H.-H.H.); a99nita32@yahoo.com.tw (H.-N.S.); 2 Mackay Medicine, Nursing and Management College, Taipei 10449, Taiwan; 3 Department of pathology, Changhua Christian Hospital, Changhua 500, Taiwan; yuemin0607@yahoo.com.tw; 4 Medical Technology, Jen-The Junior College of Medicine, Nursing and Management, Miaoli 35664, Taiwan; 5 Department of Nursing, Mei Ho University, Pingguang Road, Pingtung 912, Taiwan; x00003061@meiho.edu.tw; 6 Graduate Institute of Basic Medical Science, China Medical University, Taichung 40402, Taiwan; shibu.m.a@gmail.com (M.A.S.); bbblisiyi@hotmail.com (S.-Y.L.); 7 Tsao-Tun Psychiatric Center, Department of Health, Executive Yuan, Taipei 10058, Taiwan; shchang@ttpc.mohw.gov.tw; 8 Orthopaedic Department, Armed Forces General Hospital, Taichung 404, Taiwan; linchienchung@yahoo.com.tw; 9 Department of Surgery, School of Medicine, College of Medicine, Taipei Medical University, Taipei 110, Taiwan; rayjchen@tmu.edu.tw; 10 Department of Biotechnology, Bharathiar University, Coimbatore-641 046, India; padma.vijaya@gmail.com; 11 Graduate Institute of Chinese Medical Science, China Medical University, Taichung 40402, Taiwan; 12 Department of Health and Nutrition Biotechnology, Asia University, Taichung 41354, Taiwan

**Keywords:** hereditary non-polyposis colon cancer (HNPCC), prostaglandin E2 (PGE2), EP2 and EP4 receptors, COX-2, β-catenin

## Abstract

Metastasis is the most dangerous risk faced by patients with hereditary non-polyposis colon cancer (HNPCC). The expression of matrix metalloproteinases (MMPs) has been observed in several types of human cancers and regulates the efficacy of many therapies. Here, we show that treatment with various concentrations of prostaglandin E2 (PGE2; 0, 1, 5 or 10 μM) promotes the migration ability of the human LoVo colon cancer cell line. As demonstrated by mRNA and protein expression analyses, EP2 and EP4 are the major PGE2 receptors expressed on the LoVo cell membrane. The Phosphatidylinositol-4,5-bisphosphate 3-kinase (PI3K)/Akt cell survival pathway was upregulated by EP2 and EP4 activation. Following the activation of the PI3K/Akt pathway, β-catenin translocated into the nucleus and triggered COX2 transcription via LEF-1 and TCF-4 and its subsequent translation. COX2 expression correlated with the elevation in the migration ability of LoVo cells. The experimental evidence shows a possible mechanism by which PGE2 induces cancer cell migration and further suggests PGE2 to be a potential therapeutic target in colon cancer metastasis. On inhibition of PGE2, in order to determine the downstream pathway, the levels of PI3K/Akt pathway were suppressed and the β-catenin expression was also modulated. Inhibition of EP2 and EP4 shows that PGE2 induces protein expression of COX-2 through EP2 and EP4 receptors in LoVo colon cancer cells.

## 1. Introduction

Colorectal carcinoma (CRC) is one of the most prevalent cancers worldwide [1,2] and is the second leading cause of cancer-related mortality in developed countries [3]. Despite the availability of advanced chemotherapeutic treatments, more than 130,000 new cases of colon cancer are diagnosed per year [4], and colon cancer causes more than 56,000 deaths per year in the United States [5]. Degradation of the extracellular matrix (ECM) is closely associated with the development of malignant tumors. ECM degradation by extracellular proteinases accelerates the progress of tumor cell invasion and metastasis [6]. The proteolytic proteinase systems primarily responsible for ECM degradation in vivo are the matrix metalloproteinase (MMPs) and plasminogen activator (PA) systems [6,7]. MMPs are a family of functionally-related zinc-containing enzymes that include interstitial collagenases, gelatinases, stromelysin, matrilysin, metalloelastase, and membrane-type MMPs [8,9]. Upregulation of MMP-2 and MMP-9 has been shown to play a key role in the progression, invasion and metastasis of colorectal cancer in animal models and patients [10]. COX2 expression is markedly elevated in most human colorectal cancers [11]. Reports indicate that the COX-2 and prostaglandin E2 (PGE2) receptor subtypes are involved in intestinal carcinogenesis and activation of downstream pathways [12,13,14]. There are four specific classes of E2 receptor, EP1–4 [15]. Here, we screened these receptors (EP1, EP2, EP3 and EP4) for PGE2 activation [16]. The effect of PGE2 is induced via EP2 and EP4 receptors and results in a series of events. Phosphorylated Akt and EP1/EP4 are over-expressed in colorectal tumor tissue. Kirsten Ras (KRAS) induces HT29 cell proliferation through the expression of COX2, EP1/EP4, p-Akt and GSK3β and increases Tcf transcriptional factor activation [17]. Additionally, the Ras protein was suppressed when Ras-HT29 cells were treated with an EP4 inhibitor. In a cell-cycle assay, a K-ras mutation resulted in a prolonged S phase and an increase in the proportion of cells in G2/M phase. Furthermore, the upregulation of COX expression by the Ras/PI3K/GSK3β/β-catenin pathway is potentially responsible for the PGE2-induced migration in the human colon cancer cell line LoVo [18].

In observational studies, PGE2 induced COX2 expression, and we used receptor antagonists [17,19,20] to block the expression of PGE2, thereby inhibiting the expression of the proteins downstream of PGE2. We also used small interfering RNA (siRNA) [21] to inhibit the translation of the receptor proteins and thereby downregulate PGE2 expression. Further, we used immunoblotting and nuclear extraction assays to analyze the translocation of β-catenin from the cytosol to the nucleus, which affected the PGE2 induced COX-2 expression and the signified the role of EP2 and EP4 in PGE2 mediated signaling pathway. The results elucidate the potential for strategies and targets to control prostaglandin E2-associated tumorigenesis, proliferation and metastasis in colorectal cancers.

## 2. Results

### 2.1. Effect of PGE2 on Viability of LoVo Cells

**Figure 1:**
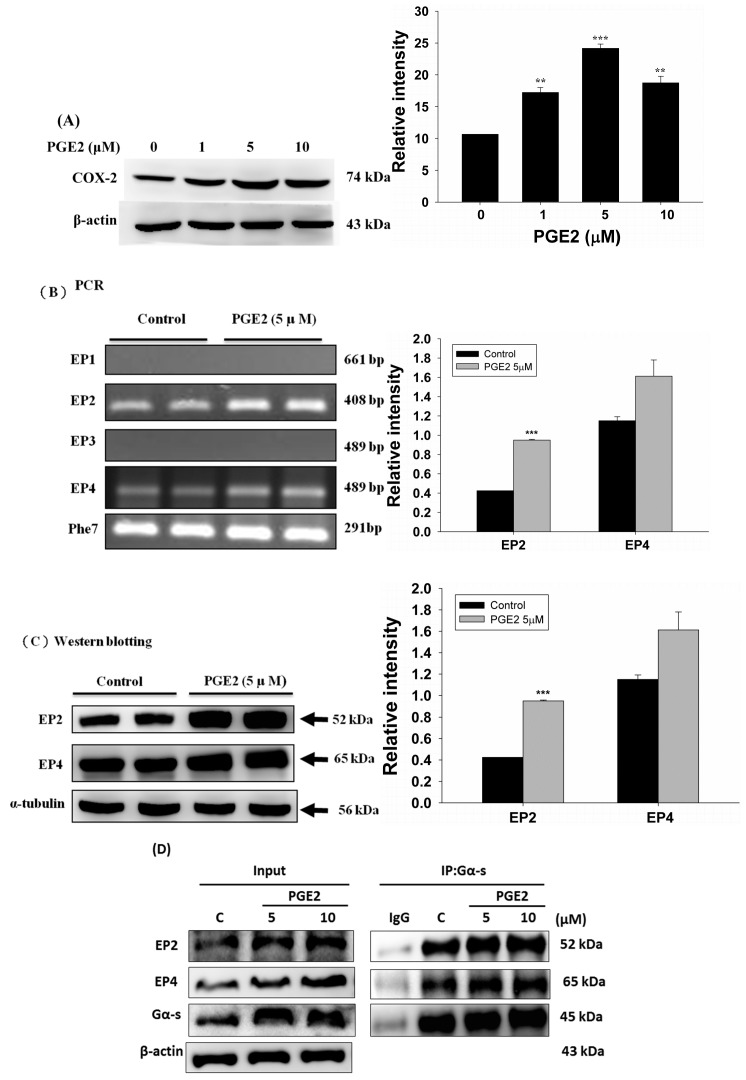
Prostaglandin E2 (PGE2) induces protein expression of COX-2 through EP2 and EP4 in LoVo cells. (**A**) LoVo cells were treated with increasing concentrations of PGE2 (0, 1, 5 and 10 μM) for 6 h, and cells were then harvested and lysed. Cell extracts were separated by 12% sodium dodecyl sulfate polyacrylamide gel electrophoresis (SDS-PAGE), transferred to polyvinylidene difluoride (PVDF) membranes, and immunoblotted with an antibody against COX-2. (**B**) LoVo cells were treated with 5 μM PGE2 for 6 h. The expression of EP1–4 in LoVo cells was detected by reverse-transcription PCR. (**C**) LoVo cells were treated with 5 μM PGE2 for 6 h. Cells were harvested and analyzed for expression of EP2 and EP4 by immunoblot. (**D**) LoVo cells were pretreated with PGE2 (5 or 10 μM) for 6 h and then harvested for immunoblot analysis using antibodies against Gα-s, EP2 and EP4. A co-immunoprecipitation assay was used to determine the degree of association of Gα-s with EP2 or EP4. An antibody against Gα-s was used for the immunoprecipitation, while antibodies against Gα-s, EP2 and EP4 were used for the immunoblot analysis. ** *p* < 0.01, *** *p* < 0.001 denotes significant differences from control values. The results were presented as the mean ± standard deviation (SD) of three difference experiments.

The expression of COX2 in LoVo cells was evaluated by immunoblot assay and was found to increase following treatment with PGE2 at various concentrations (0, 1, 5 and 10 μM) (Figure 1A). Analysis of the mRNA expression of the PGE2 downstream receptors (EP1, EP2, EP3 and EP4) by PCR assay shows that EP2 and EP4 are the major PGE2 targets on LoVo cell membranes (Figure 1B). Similarly, the expression of the EP2 and EP4 proteins also increased within 6 h of treatment with 5 μM PGE2 (Figure 1C). Immunoblotting assay results revealed that the degree of association of Gα-s with EP2 or EP4 increased following treatment with 5 of 10 μM PGE2 (Figure 1D).

### 2.2. PGE2 Treatment Upregulated the Expression of COX2 and Proteins in the PI3K/Akt and p-GSK3β/β-Catenin Pathways

**Figure 2:**
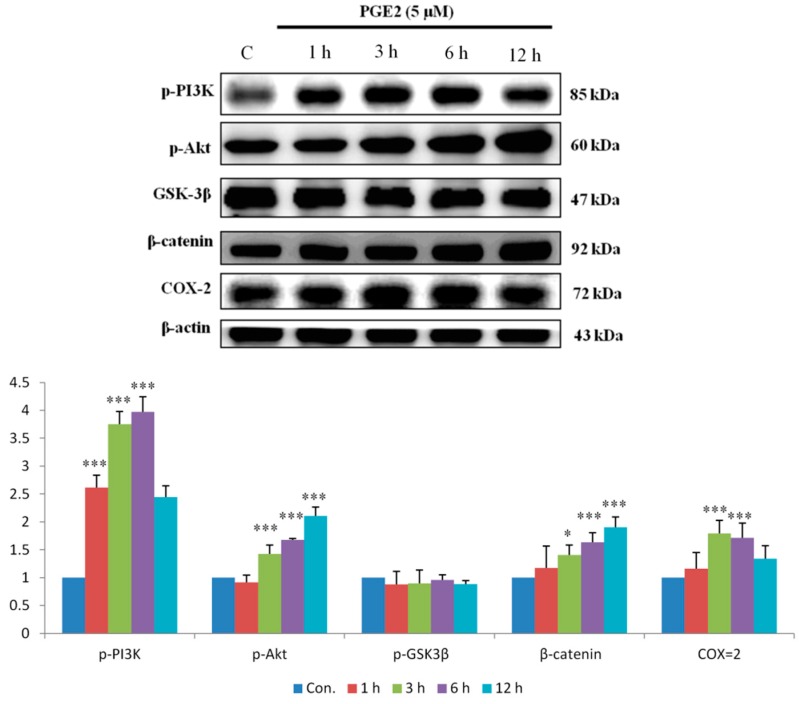
PGE2 promotes the expression of Phosphatidylinositol-4,5-bisphosphate 3-kinase PI3K, Akt, p-GSK3β, β-catenin and COX-2 proteins in LoVo cells. LoVo cells were treated with 5 μM PGE2 for 1, 3, 6 and 12 h. Cells were harvested and lysed at the indicated time points. Cell extracts were separated by 12% SDS-PAGE, transferred to PVDF membranes, and immunoblotted with antibodies against PI3K, Akt, p-GSK3β, β-catenin and COX2. The expression of these downstream proteins exhibited an increasing trend with longer PGE2 treatment times. * *p* < 0.05, *** *p* < 0.001 denotes significant differences from control values. The results were presented as the mean ± SD of three difference experiments.

EP2 and EP4 are also major proteins upregulated by the PI3K/Akt pathway. As the results show, PI3K and Akt were activated within 1 h of PGE2 treatment. The downstream proteins p-GSK3β and β-catenin were also activated 6 to 12 h after treatment. Finally, PI3K/Akt pathway activation increased COX2 expression (Figure 2).

### 2.3. EP2- or EP4-Specific siRNA and Receptor Antagonists Inhibit the Induction of PI3K, Akt, β-Catenin and COX2 in PGE2-Treated LoVo Cells

**Figure 3:**
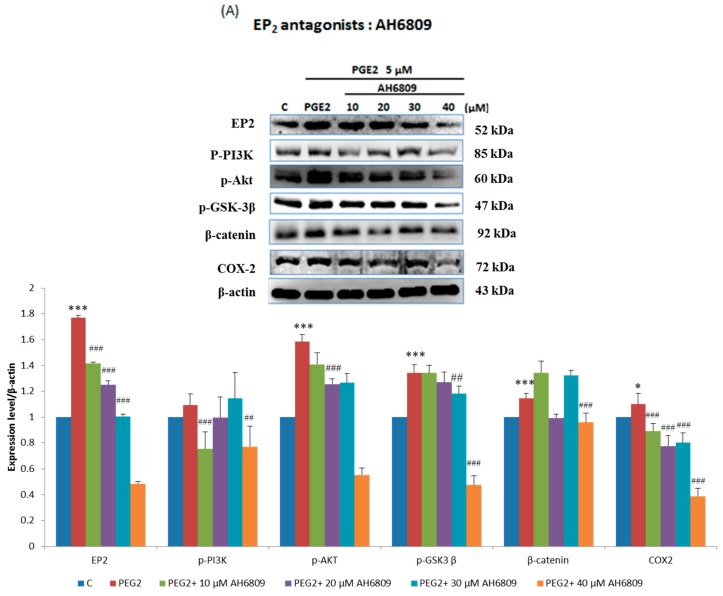

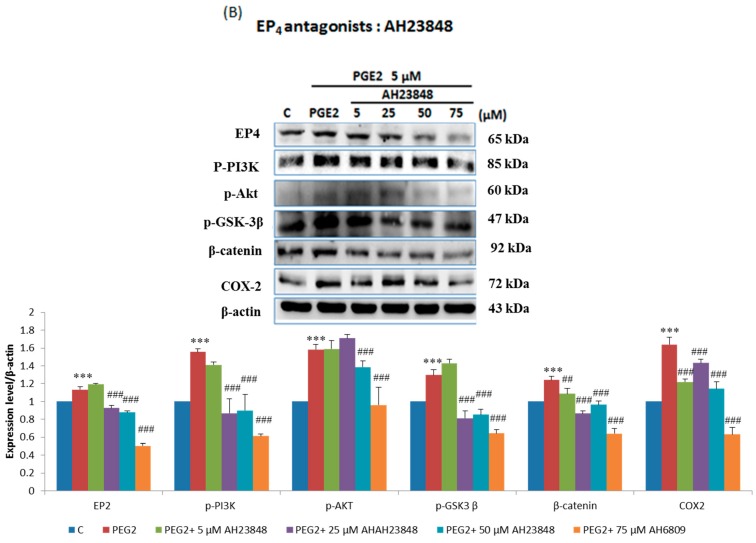

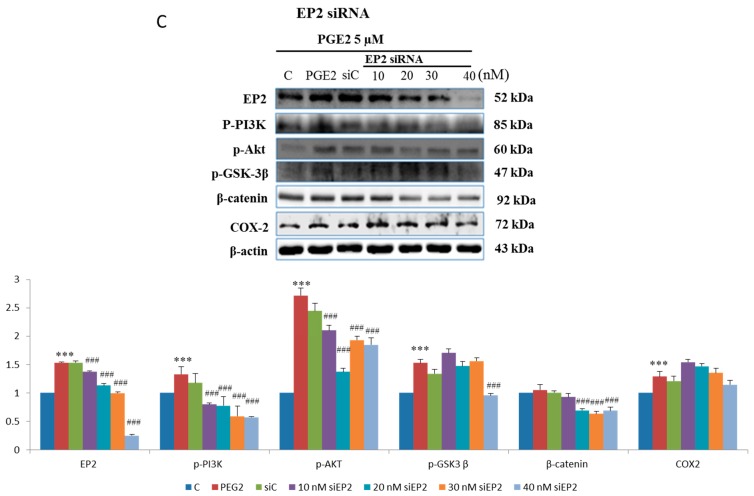

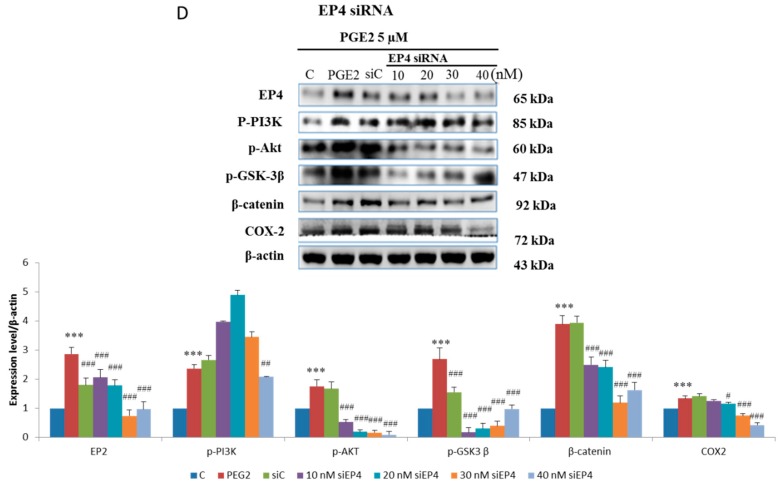
Effect of inhibitors on protein expression in PGE2-treated LoVo cells. LoVo cells were pretreated with either (**A**) the EP2 antagonist AH6809 or (**B**) the EP4 antagonist AH23848 for 21 h and then treated with 5 μM PGE2 for 24 h, or pretreated with either (**C**) EP2 small interfering RNA (siRNA) or (**D**) EP4 siRNA for 42 h and then treated with 5 μM PGE2 for 48 h. Cells were then harvested and analyzed by immunoblot using antibodies against p-PI3K, p-Akt, p-GSK3β, β-catenin and COX2 to determine the effect of PGE2 inhibition on protein expression. * *p* < 0.05, *** *p* < 0.001 denotes significant differences from control values ^#^ *p* < 0.05, ^##^ *p* < 0.01 and ^###^ *p* < 0.001 denote significant differences when compared to PGE2-treated groups. The results were presented as the mean ± SD of three difference experiments.

In order to verify this pathway LoVo cells were pretreated with (Figure 3A) EP2 antagonists: AH6809 (10, 20, 30, 40 μM) and (Figure 3B) EP4 (5, 25, 50, 75 μM) antagonists: AH23848; These antagonists competed with PGE2, therefore the activation of the receptor was suppressed and down-regulated the downstream pathway. To identify whether EP2 and EP4 were involved in COX2 expression, we applied small interfering RNA (siRNA)-mediated specific knockdown of EP2 and EP4 in human LoVo colon cancer cells (Figure 3C,D). LoVo cells transfected with scramble siRNA (10 nM) EP2 siRNA and EP4 siRNA (10, 20, 30, 40 nM) for 48 h were treated with PGE2 (5μM) for 6 h. As the results show, specific EP2 and EP4 siRNA significantly reduced the protein level of EP2 and EP4 receptors in LoVo cells. According to the results of the western blot assay, the PI3K/Akt survival pathway was not significantly expressed and β-catenin was degraded in a dose-dependent manner.

### 2.4. Immunofluorescence Assays and Analysis of Cytosolic and Nuclear Protein Fractions

**Figure 4:**
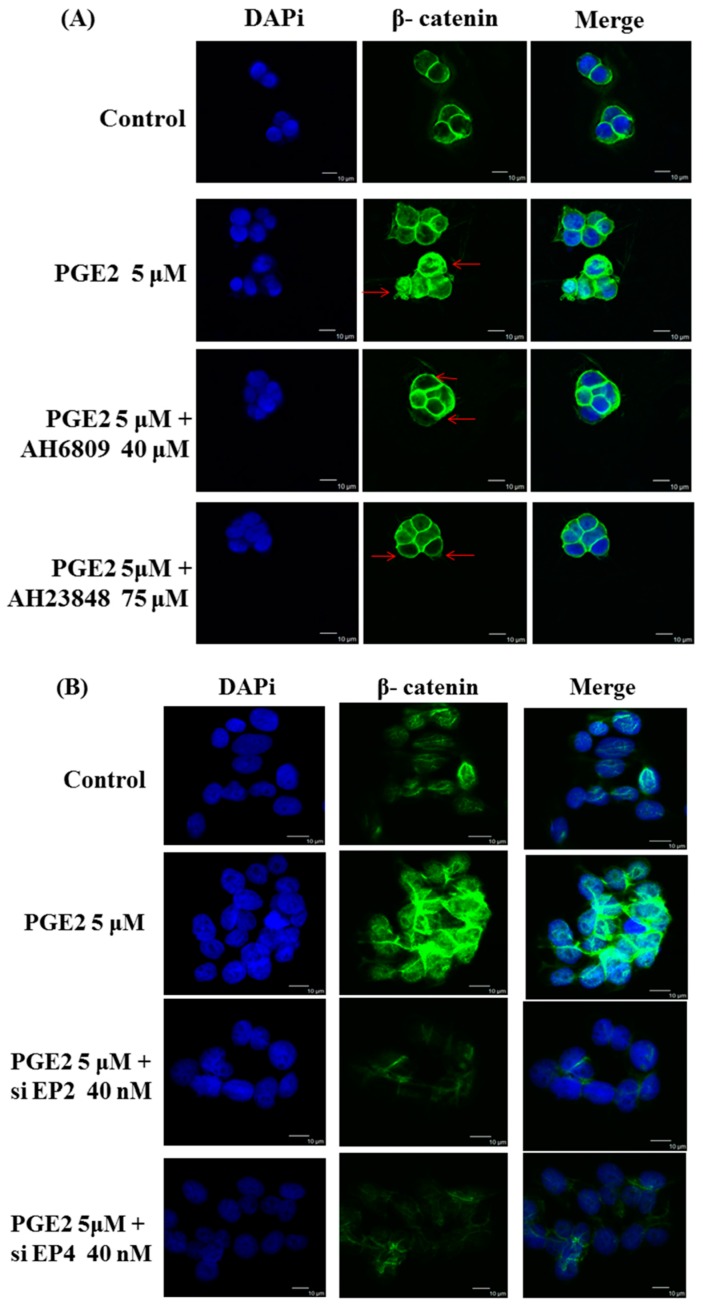

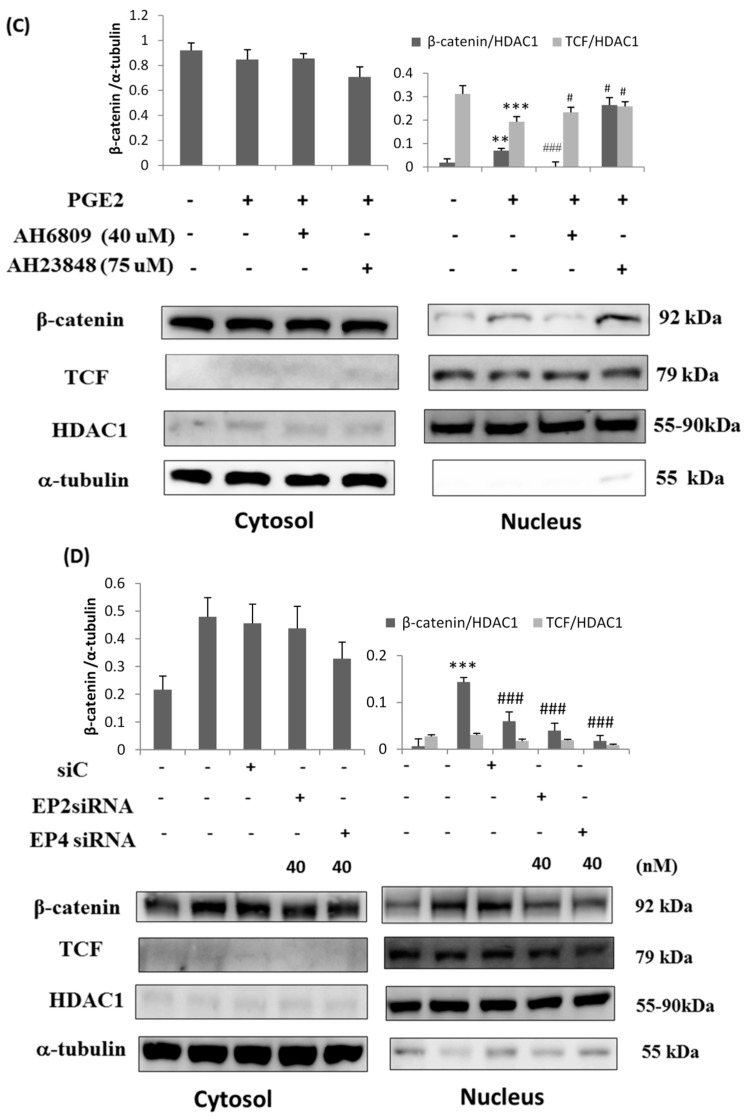
Prostaglandin E2 (PGE2) treatment caused the translocation of β-catenin from the cytosol to the nucleus in LoVo cell. LoVo cells were pretreated with (**A**) the EP2 antagonist AH6809 or the EP4 antagonist AH23848 or (**B**) EP2 siRNA or EP4 siRNA, and then treated with PGE2 (5 μM). Immunofluorescence assays were performed in LoVo cells using an antibody against β-catenin (1:250, green stain, Red arrows) followed by 4′,6-diamidino-2-phenylindole DAPI nuclear counterstaining (blue). The merged images of β-catenin (green) with DAPI (blue) are also shown. In addition, the cytosolic and nuclear proteins were isolated from LoVo cells pretreated with (**C**) the EP2 antagonist AH6809 or the EP4 antagonist AH23848 or (**D**) EP2- or EP4-specific siRNA and treated with PGE2 (5 μM). The expression of β-catenin, LEF-1, TCF-4 and HDAC1 was detected by immunoblot assay. ** *p* < 0.01 and *** *p* < 0.001 denotes significant differences from control values ^#^ *p* < 0.05 and ^###^ *p* < 0.001 denote significant differences when compared to PGE2 treated groups. The results were presented as the mean ± SD of three difference experiments.

Immunofluorescence assays were performed in LoVo cells pretreated with the EP2 antagonist AH6809 (40 μM), the EP4 antagonist AH23848 (75 μM), EP2-specific siRNA (40 nM) or EP4-specific siRNA (40 nM) and treated with PGE2 (5 μM) using an anti-β-catenin antibody (1:250, green) followed by 4′,6-diamidino-2-phenylindole DAPI nuclear counterstaining (blue). The confocal microscopy images show that PGE2 treatment caused β-catenin to translocate into the nucleus in LoVo cells. In addition, the translocation of β-catenin was controlled by PGE2 antagonist treatment (Figure 4A,B). The nuclear and cytosolic proteins were separated and analyzed by immunoblot assay. Two COX2-promoting proteins, LEF-1 and TCF-4, were located in the nucleus and were upregulated by PGE2 treatment in a dose-dependent manner. In addition to LEF-1 and TCF-4, PGE2 treatment also caused β-catenin to translocate into the nucleus (Figure 4C,D).

### 2.5. Inhibition of PGE2-Induced Cell Migration by Antagonist and siRNA Treatment

The migration of the PGE2-treated LoVo cells was determined by a migration assay.

**Figure 5:**
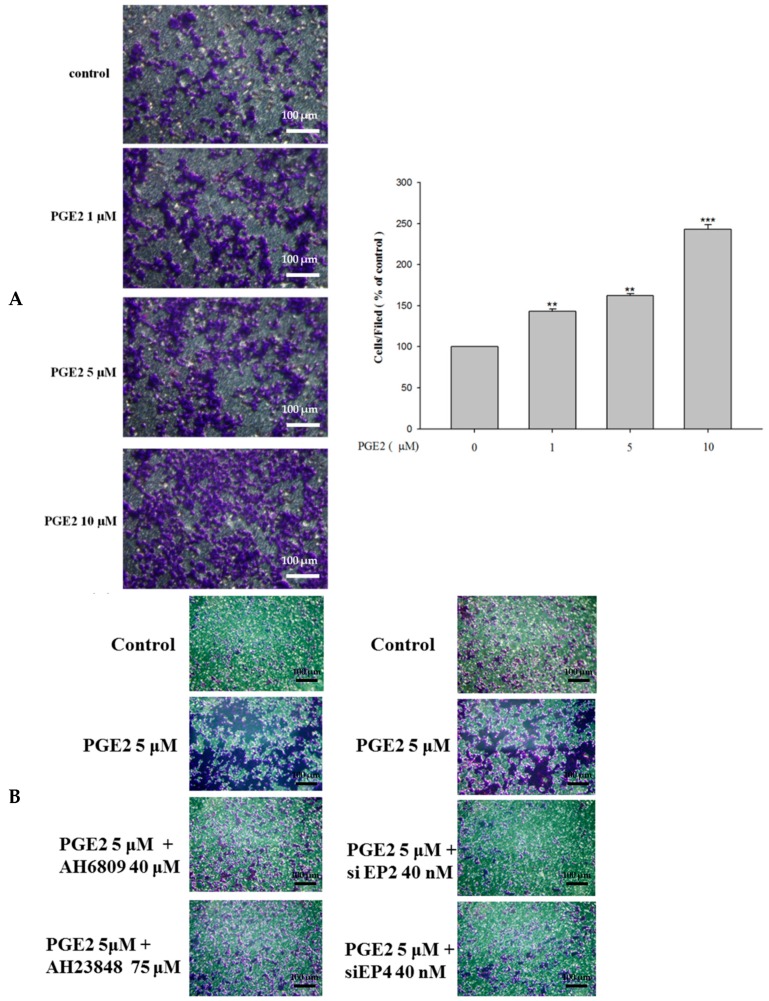
PGE2 can efficiently promote the migration ability of LoVo cells. (**A**) LoVo cells were pretreated with increasing concentrations of PGE2 (0, 1, 5 and 10 μM) for 6 h. Cells were harvested and migration ability was subsequently evaluated by a migration assay. (**B**) The use of antagonists or siRNA reduces the migration ability of LoVo cells. The responses to different treatments were observed and analyzed with a fluorescence microscope. *** *p* < 0.001 versus control. **, *p* < 0.01 versus control (mean ± SD, *n* = 3).

As the results show, PGE2 treatment (0, 1, 5 or 10 μM) efficiently promoted the migration abilities of LoVo cells (Figure 5A). Additionally, blocking EP2 and EP4 by antagonist treatment or knocking down their expression by siRNA treatment significantly blocked the PGE2-induced effects on LoVo cell migration (Figure 5B). EP2 and EP4 antagonists or siRNA treatment also significantly blocked the effects of PGE2 in cell migration of LoVo cells [22].

## 3. Discussion

Inflammation is emerging as a new hallmark of cancer metastasis and invasion and cyclooxygenases (COXs) and lipoxygenase (LOX) play major roles in arachidonic acid-related inflammatory cascades [23,24]. Previous studies show that COX-2 as a rate-limiting enzyme involved in the formation of PGE2 from arachidonic acid, and is over-expressed in the colorectal cancer tumor tissues. Elevated levels of COX-2 are often correlated with colorectal tumorigenesis and progression by promoting tumor cell proliferation and inhibiting apoptosis, tumor angiogenesis, and tumor cell attachment, facilitating metastasis [25]. COX-2 expression in colorectal cancers is 80–90% higher than in the normal tissue. Other cancers such as those of the head, breast, cervix, bladder and gastrointestinal system have also exhibited high levels of COX-2 expression [26]. Various epidemiological and clinical studies have shown 40–50% reduction in colorectal cancer risks in humans administered with non-steroidal anti-inflammatory drugs. Selective COX-2 inhibitors has demonstrated anti-cancer activities in vivo and in vitro and accumulating evidence shows that small-molecule inhibitors targeting COX-2 could hinder the occurrence of colorectal cancer [27,28,29,30].

Hereditary colon cancers include familial adenomatous polyposis (FAP) and hereditary non-polyposis colon cancer (HNPCC) that accounts for more than 5 percent of all cases of colorectal cancer [31,32,33]. Surgical removal is the only definite way to prevent colon cancer because patients diagnosed with HNPCCs are at a high risk for synchronous metastasis through the lymphatic system [34,35]. PGE2 and EP (1–4) receptors play a leading part in angiogenesis, with the best effect in tumor cells and endothelial cells. PGE2 is involved in diseases that affect the tissue, and is correlated with the expression of mRNA of the prostaglandin receptors EP3 and EP4 [34,35]. EP1 to EP4 receptors are differentially expressed in other cancers. In endometriosis patients, the expression of EP3 and EP4 increased only in the ectopic endometrium and the effect of EP1 or EP2 receptor expression was not observed [36,37]. Differential expression of EP receptors in different cell models is a known phenomenon and previous reports also show that human U373 MG astrocytoma cells and rat primary astrocytes show evidence of total dormant expression in some human lines [38]. Therefore, screening before the initiation of cancer cell migration is necessary. PGE2 is known to activate the EP1, EP2, EP3 and EP4 receptors, leading to cell migration through the downstream COX-2 expression. EP2 and EP4 are two of the major receptors in LoVo cells. Co-immunoprecipitation results showed that the degree of association of Gα-s with EP2 or EP4 increased after treatment with PGE2 in LoVo cells. In the four EP receptors, both EP2 and EP4 are G-linked Therefore, its activation leads to increased intracellular activation of PKA after cAMP [39] and besides, EP4 is activated can also stimulate the ERK [40] and PI3K/Akt [41] pathways, and promote PGE-2-dependent migration [42] and cell survival [43]. Our experiments show that EP2 and EP4 were the major proteins involved in upregulating the PI3K/Akt pathway after PGE2 treatment, and this survival pathway can control the p-GSK3β/β-catenin pathway.

PGE2 levels are generally at higher concentrations in tumor tissues when compared to the normal tissues [44]. PGE2 mediates tumor survival by inhibiting apoptosis of the tumor cells and induces cell proliferation [45]. Moreover, PGE2 alters the tumor cell morphology and enhances the metastatic events and thereby increases tumor progression [46,47]. In addition to the direct effects of PGE2 on tumor cells, this lipid mediator induces the production of metastasis-promoting matrix metalloproteinases and stimulates angiogenesis [48].

To prove that the receptors influence downstream proteins, we used receptor antagonists to block the interaction of PGE2 with EP2 and EP4 and thereby reduce the activation of the downstream proteins. The PGE2 receptor antagonists significantly suppressed the expression of the downstream proteins. Further, when the receptors were inhibited the downstream pathway was also suppressed. It is unclear what role the EP receptor plays in supporting smooth muscle cells and pericytes in the tumor vascular system. Targeting EP receptors can decrease the potential for angiogenesis in one cell type, but may have an adverse effect on other cell types and the entire organ system [35,36]. We use siRNA to suppress the expression of the PGE2 receptors, which resulted in decreased expression of proteins such as p-PI3K, p-Akt, p-GSK3β, β-catenin and COX2. We also determined the localization of β-catenin. When β-catenin is translocated into the nucleus of a cell, it modulates the transcription and translation status of COX-2 and regulates various genes involved in proliferation and migration of cells [49,50].

Both immunofluorescence assays and analysis of the nuclear and cytosolic protein fractions were used to determine the localization of β-catenin. The translocation of β-catenin from the cytosol to the nucleus also plays a role in the transcription and translation of COX2. Our results indicated that a decrease in the expression of COX2 results in a corresponding reduction in cell migration. LoVo cells were pretreated with PGE2 and harvested and subsequently their migration ability was determined by migration assay. We used receptor antagonists and siRNA-mediated knockdown of the receptors to determine if COX2 inhibition affected cell migration. In this study, we showed that PGE2 treatment can activate the expression of COX2, β-catenin and the PI3K/Akt pathway. Through the receptor antagonists and specific siRNAs, we demonstrated here that the PGE2 receptors EP2 and EP4 play an important role in these pathways. PGE2 suppresses apoptotic effect by mediating the IGF-II/IGF-I receptor signaling-induced PI3k/Akt activation and by increasing the ability of Ras and PI3K association which is dependent on the activation of EP4 receptor [51]. PGE2 induces the cellular events via EP2 and EP4 receptors and subsequently regulates the survival pathway; therefore, these receptors could they can be used as targets for future treatment strategies.

In canonical Wnt signaling, phosphorylation of β-catenin at specific locations by glycogen synthase kinase-3β (GSK-3β) leads to ubiquitination and degradation of β-catenin [49,52]. COX-2 and β-catenin have been linked in colon cancer but the available knowledge on their mechanism of action is limited and there is very little information available about the association between inflammatory mediators and β-catenin in colon metastasis, and therefore further exploration is needed. Further investigation on the synergistic effects of drugs against these investigated targets along with existing chemotherapeutic and molecular targeted therapies would be interesting.

## 4. Materials and Methods

### 4.1. Cells, Antibodies, Reagents and Enzymes

The human colon cancer cell line LoVo was obtained from the American Type Culture Collection (ATCC) (Rockville, MD, USA). LoVo cells were established from a metastatic nodule resected from a 56-year-old colon adenocarcinoma patient.

We utilized antibodies against the following proteins: phospho-PI3K, phospho-Akt, COX2, GSK3β, β-catenin, LEF-1 and HADAC-1 (Santa Cruz Biotechnology, Inc., Santa Cruz, CA, USA); EP-2 and EP-4 (Cayman Chemical Company, Ann Arbor, MI, USA); and TCF-4 (Cell Signaling Technology, Inc., Beverly, MA, USA). Antibodies to α-tubulin or β-actin (Santa Cruz Biotechnology, Inc., Santa Cruz, CA, USA) were used as loading controls. The goat anti-mouse IgG, goat anti-rabbit IgG and rabbit anti-goat IgG antibodies, all conjugated to horseradish peroxidase, were purchased from Santa Cruz Biotechnology, Inc. in California, USA.

### 4.2. Cell Culture

LoVo colon cancer cells from the American Type Culture Collection (ATCC) (Rockville, MD, USA) were cultured in 10 cm^2^ culture dishes in Dulbecco’s modified Eagle’s medium (DMEM) supplemented with 100 U/mL penicillin, 100 μg/mL streptomycin, 2 mM glutamine, 1 mM 4-(2-hydroxyethyl)-1-piperazineethanesulfonic acid (HEPES) buffer, and 10% Clontech fetal bovine serum in humidified air (5% CO_2_) at 37 °C. LoVo cell passpage were re-plated at a density of 1 × 10^5^ cells in 6 cm^2^ culture dishes.

### 4.3. Total RNA Extraction and Reverse Transcription

Total RNA was extracted using the Ultraspec RNA Isolation System (Biotecx Laboratories, Houston, TX, USA) according to the manufacturer’s directions. LoVo cells were thoroughly homogenized (1 mL Ultraspec reagent) with a homogenizer in a polypropylene tube, and then total RNA was isolated using a standard method. The RNA precipitate was washed twice by gentle vortexing with 70% ethanol, collected by centrifugation at 12,000× *g*, dried under vacuum for 10 min, dissolved in 50 μL of diethylpyrocarbonate-treated water, and incubated for 15 min at 60 °C. The RNA was quantified and checked for purity by spectrophotometry at a wavelength of 260 nm/280 nm. An aliquot of total RNA (0.5 μg) was reverse transcribed using 0.5 μM oligo d(T) primers in a reaction solution (50 μL) containing 75 mM KCl, 50 mM Tris–HCl (pH 8.3), 3 mM MgCl_2_, 10 mM dithiothreitol (DTT), RNase inhibitor (Promega, Madison, WI, USA), 0.8 mM total deoxynucleotide (dNTPs), and 200 U of Moloney murine leukemia virus reverse transcriptase (Promega). The sample was incubated at 42 °C for 1 h and at 95 °C for 5 min before being chilled on ice for 10 min.

Primers were as follows: human EP-1 forward primer CTGGGCGGCTGCATGGTCTTCTTC, reverse primer AGTGGCCGCTGCAGGGAGGTAG; human EP-2 forward primer CGAGACGCGACAGTGGCTTCC, reverse primer CGAGACGCGGCGCTGGTAGA; human EP-3 forward primer CGGGGCTACGGAGGGGATGC, reverse primer ATGGCGCTGGCGATGAACAACGAG; human EP-4 forward primer TCGCGCAAGGAGCAGAAGGAGAC, reverse primer GACGGTGGCGAGAATGAGGAAGGA.

#### The Expression of EP1–EP4 in LoVo Colon Cancer Cells Was Detected by Reverse Transcription PCR (RT-PCR)

RT-PCR was carried out with the Ex Taq polymerase using Ex Taq Master Mix kit (Clontech Laboratries, Mountain View, CA, USA). One microliter of reverse transcription reaction was used for quantitative PCR in a total volume of 25 µL. RT-PCR was performed using 95 °C for 10 min followed by 40 cycles of 95 °C for 15 s, 53 °C for 15 s and 72 °C for 10 s.

### 4.4. Immunoblotting Assay

To isolate total proteins, cultured LoVo cells were washed with cold PBS and resuspended in lysis buffer (50 mM Tris, pH 7.5, 0.5 M NaCl, 1.0 mM ethylenediaminetetraacetic acid (EDTA), pH 7.5, 10% glycerol, 1 mM β-mercaptoethanol, 1% IGEPAL-630 and a proteinase inhibitor cocktail (Roche Molecular Biochemicals, Basel, Switzerland). After a 30-min incubation on ice, the supernatant was collected by centrifugation at 12,000× *g* for 15 min at 4 °C, and the protein concentration was determined by the Bradford method. Samples containing equal amounts of protein (60 μg) were analyzed by immunoblot. Briefly, proteins were separated by 12% SDS-PAGE and transferred onto PVDF membranes (Millipore, Belford, MA, USA). The membranes were blocked with blocking buffer (5% non-fat dry milk, 20 mM Tris-HCl, pH 7.6, 150 mM NaCl, and 0.1% Tween 20) for at least 1 h at room temperature. Membranes were incubated in 1:1000 primary antibodies (EP2, EP4, COX-2, α-Tubulin, β-actin, Gαs, p-Akt, p-PI3K, GSK-3β, β-Catenin, Cayman Chemical, Santa Cruz Biotechnology, Cell Signaling) in the above solution on an orbital shaker at 4 °C overnight. Following the primary antibody incubations, membranes were incubated with horseradish peroxidase-linked secondary antibodies (anti-rabbit, anti-mouse, or anti-goat IgG) and soaked and rocked in 1:1000 secondary antibody (Santa Cruz Biotechnology) solutions for 1 h at room temperature. The signal detection was performed using enhanced chemiluminescence (ECL) reagent and a digital imaging system.

### 4.5. Co-Immunoprecipitation Assay (Co-IP)

LoVo colon cancer cells were treated with PGE2. Cells were lysed for 30 min on the culture plates in 200 μL Co-IP cell lysis buffer (1.5 mM MgCl_2_, 1% Triton X-100, 50 mM HEPES, 1 mM EDTA, 150 mM NaCl, 10% glycerol, 1 mM NaVO3, 10 mM NaF, 10 mM β-glycerophosphate, and 5 mg/mL protease inhibitor). The cells were then scraped and centrifuged for 10 min at 12,000× *g* at 4 °C. For each group, 100 μg of the total protein sample was added into each microcentrifuge tube with enough lysis buffer, without protease inhibitor added, to bring the final volume to 500 μL. A total of 7 μL of Gα-s antibody (K-20) (Santa Cruz Biotechnology, Inc., cat. no. sc-823) was then added, followed by vortex at 4 °C for 1 h with a vortex mixer. The samples were then centrifuged at 4 °C and 1300× *g* for 30 s. The supernatant was then removed. For each group, 2.5 μL of a Gα-s antibody was added to each of the microcentrifuge tubes. The microcentrifuge tubes were vortexed at 4 °C overnight with a vortex mixer. An aliquot of 20 μL of Protein G PLUS Agarose was added, followed by vortexing at 4˚C for 2 h with the vortex mixer and then centrifuged at 4 °C and 1300× *g* for 30 s. After the supernatant was removed, 1 mL of lysis buffer without protease inhibitors was added to wash the pellet, followed by centrifugation at 4 °C and 1300× *g* for 30 s. Western blot analysis was performed to detect the Gα-s, EP2 and EP4 proteins.

### 4.6. Gene Knockdown Using siRNA

LoVo colon cancer cells were seeded into 15 cm^2^ culture dishes and grown to 80% confluency. The siRNA transfections were performed using DharmaFECT Duo transfection reagent (Dharmacon, Inc., Lafayette, CO, USA). The siRNAs specific to EP2 and EP4 (Santa Cruz Biotechnology, Inc.) and the negative control Non-Targeting Pool (NT) (Dharmacon, Inc.) were mixed with serum-free medium. The detailed procedure has been described in our previous study.

### 4.7. Migration Assay

The migration assay was performed using a density of 5 × 10^4^ LoVo cells/well cultured in a 48-well Boyden chamber plate (Neuro Probe, Gaithersburg, MD, USA) and polycarbonate membrane filters with a pore size of 8 µm. The lower compartment was filled with DMEM containing 20% FBS. LoVo cells were placed in the upper part of the Boyden chamber with serum-free medium and incubated for 48 h. After incubation, the cells on the membrane filter were fixed with methanol and stained with 0.05% Giemsa for 1 h. The cells on the upper surface of the filter were removed with a cotton swab. The filters were then rinsed in double-distilled water until any additional stain was leached. The cells were then air-dried for 20 min. The migratory phenotypes were determined by counting the cells that had migrated to the lower side of the filter with microscopy at 200× and 400× magnification. Ten fields were blindly selected and counted as the mean cell number in each filter, and each sample was assayed in triplicate.

### 4.8. Nuclear Extraction

Cytosolic and nuclear proteins were isolated using an extraction reagent-containing membrane lysis buffer (10 mM HEPES (pH 8.0), 1.5 mM MgCl_2_, 10 mM KCl, 1 mM DDT, and proteinase inhibitor) and nuclear lysis buffer (20 mM HEPES (pH 8.0), 1.5 mM MgCl_2_, 10 mM NaCl, 1 mM DDT, 0.2 mM EDTA, 0.25 M glycerol, and proteinase inhibitor). In brief, following the treatments, cells were resuspended in PBS and membrane lysis buffer was added. After a 10-min incubation on ice, the cells were centrifuged at 12,000 rpm for 2 min to pellet the nuclei. The supernatant was stored for use as the cytosolic fraction, and the nuclei pellet was lysed with nuclear lysis buffer to obtain the nuclear fraction.

### 4.9. Immunofluorescence Assay

LoVo cells were treated with increasing concentrations of PGE2 dosage (0, 1, 5 and 10 μM) for 6 h. The cells were fixed by 4% paraformaldehyde and the immunofluorescence assay was performed in LoVo cells using an antibody against β-catenin (1:250, green) followed by DAPI nuclear counterstaining (blue), and the results were analyzed by confocal microscopy. Merged images of β-catenin (green) with DAPI (blue) were obtained using confocal microscope (Bruker, Billerica, MA, USA).

### 4.10. Statistical Analysis

Each experiment was repeated in triplicates. The results were presented as the mean ± SD, and statistical comparisons were made using one way ANOVA. Significance was defined at the *p* < 0.05 or 0.01 levels.

## 5. Conclusions

EP2 and EP4 mediate the PGE2-induced COX2 expression and cell migration in LoVo colon cancer cells correlated with COX2 expression. PGE2 interaction with the EP2 and EP4 receptors results in upregulation of the PI3K/Akt pathway, and this survival pathway can control the p-GSK3β/β-catenin pathway. In addition, β-catenin also translocates into the nucleus and interacts with LEF-1 and TCF-4 at the COX2 promoter to activate the transcription of COX2.
